# Special Issue: Novel Antifungal Drug Discovery

**DOI:** 10.3390/jof2040033

**Published:** 2016-12-14

**Authors:** Maurizio Del Poeta

**Affiliations:** 1 Department of Molecular Genetics and Microbiology, Stony Brook University, Stony Brook, NY 11794, USA; maurizio.delpoeta@stonybrook.edu; Tel.: +1-631-632-4024; Fax: +1-631-632-9797; 2 Veterans Affairs Medical Center, Northport, NY 11768, USA

**Keywords:** antifungal drug, fungal infection, fungal virulence, sphingolipid, phospholipase, drug resistance, mathematical model

## Abstract

This Special Issue is designed to highlight the latest research and development on new antifungal compounds with mechanisms of action different from the ones of polyenes, azoles, and echinocandins. The papers presented here highlight new pathways and targets that could be exploited for the future development of new antifungal agents to be used alone or in combination with existing antifungals. A computational model for better predicting antifungal drug resistance is also presented.

Globally, over 300 million people are afflicted by a serious fungal infection and 25 million are at risk of dying or losing their sight [1]. Among fungal infections, invasive infections such as cryptococcosis, candidiasis, aspergillosis and pneumocystosis are the most common and the most life-threatening [2,3,4]. These infections have risen dramatically over the last 30 years, some, such as invasive aspergillosis, over 14-fold in certain hospital setting [5]. The Center for Disease Control and Prevention (CDC) estimates that more than one million new cases per year of cryptococcosis will occur worldwide in patients with AIDS, and ~600,000 will die from the infection [6]. This is a drastic increase considering that prior to the mid-1950s, fewer than 300 cryptococcosis cases had been reported in the medical literature (reviewed in [7]). Certain medical devices, such as catheters, provide the port of entry to fungi that colonize the skin and mucosa. As a result, disseminated candidiasis is the fourth most common hospital-acquired sepsis [8,9,10] with an estimate of >120,000 deaths/year [2,11]. Another invasive fungal infection, disseminated aspergillosis, is steadily increasing in immunocompromised patients [12,13,14,15] with a mortality rate of 40%–90% [2], resulting in an estimate of 450,000 deaths/year, according to the Leading International Fungal Education (LIFE) [11]. *Aspergillus* spp. is also responsible for severe asthma by fungal sensitization (SAFS), accounting for an estimate of annual deaths of 100,000 [16].

*Pneumocystis* spp. are a group of host-specific opportunistic fungi that reside in the lungs of humans and animals in nature. *Pneumocystis* pneumonia (PCP) remains the most prevalent opportunistic infection in patients infected with the human immunodeficiency virus (HIV). Reports on mortality rates for PCP are variable, ranging from 13% to as high as 80%, which even at the lowest rate results in more than 52,000 deaths per year [17]. PCP is also prevalent in other patient groups, notably patients who are chronically immune suppressed due to solid organ transplantation or due to chemotherapy for cancer or autoimmune disease. In addition to being the cause of PCP in immunocompromised hosts, *P. jirovecii* (Pj) is also a frequent colonizer of the respiratory tract in immunocompetent individuals with other underlying pulmonary diseases, such as Chronic Obstructive Pulmonary Disease (COPD), in which it initiates a deleterious inflammatory reaction [18].

Based on these reports, over 1,300,000 people are estimated to die every year because of invasive fungal infections. This mortality is similar to the one from malaria (1,240,000/year) [19] and tuberculosis (1,400,000/year) [20]. Most likely, the mortality caused by fungal infections is an underestimated figure as these infections are not as widely reportable as malaria and tuberculosis.

Because of this alarming increase, two important programs started in 2013: the Global Action Fund for Fungal Infections (GAFFI) and the Leading International Fungal Education (LIFE). The main missions of these programs are to reduce illness and death associated with fungal diseases worldwide and to educate health professionals around the world on the high morbidity and mortality of invasive fungal infections [16] (please visit www.gaffi.org).

While there are about 30 branded prescription antifungal drugs on the market, three classes of antifungals are mainly used to manage all types of invasive fungal infections: (1) azoles, such as fluconazole, launched in the mid-1980s; (2) polyenes, such as amphotericin B, launched in the mid-1950s; and (3) echinocandins, such as caspofungin, launched in early 2000. The market for antifungals has historically been dominated by the large pharmaceutical companies, including Pfizer, J&J/Ortho McNeil/Janssen, Merck, Novartis, Astellas, Schering Plough (now part of Merck), GlaxoSmithKline, and BristolMyersSquibb. In recent years, however, these pharmaceutical companies have discontinued or significantly decreased their investment in the R&D of new antifungals. At the same time, the increased use of current azoles has led to an increase in drug resistance, limiting their effectiveness. Drug–drug interaction issues are a major impediment to the use of the azoles voriconazole, itraconazole and posaconazole. The interactions with cancer chemotherapy agents and immunosuppressants are particularly difficult to handle clinically. Systemic antifungals such as amphotericin B tend to have relatively high toxicity and side effects even when combined with Flucytosine. The echinocandins have a lower incidence of adverse events compared to older antifungals but they bind highly to serum proteins, there are no oral formulations, and their antifungal spectrum of activity is very narrow [21,22,23,24,25,26]. There is a need for antifungal compounds that are more effective and safe versus current antifungals. Pharmaceutical companies are looking to small biotech companies and academia to supply the next generation of antifungal compounds due to their own decreasing investment in antifungal R&D.

In the case of *Pneumocystis*, the situation is direr. *Pneumocystis* pneumonia (PCP) does not respond to any of the standard antifungals described above [27,28]. The drug of choice for treatment and chemoprophylaxis of PCP is trimethoprim-sulfamethoxazole (TMP-SMX). Analysis of *P. jirovecii* isolates demonstrates that the pathogen is evolving mutations in the target genes of TMP-SMX, suggesting *P. jirovecii* could soon become resistant to SMX in the combination, which is considered the more potent of the two drugs that make up the combination therapy [29]. Atovaquone and pentamidine, both second-line treatments, suffer from low efficacy and severe adverse events that include nephrotoxicity, neutropenia, hypotension and hypoglycemia [30]. Atovaquone inhibits the mitochondrial cytochrome Bc1 complex in parasites at much lower concentrations than the respective mammalian complex. However, evolving resistance to atovaquone, corresponding to mutations in the *Pneumocystis* cytochrome *b* gene, has been observed [31]. Pentamidine has a broad antimicrobial action with no specific known target and is highly toxic and often considered to be a drug of last resort. We are faced, then, with a growing patient population, a microorganism that cannot be easily subjected to detailed biochemical analysis in the laboratory, a developing resistance to standard-of-care medications and a limited industrial effort to advance new therapies into the clinic.

In this Special Issue entitled “Novel Antifungal Drug Discovery”, the paper by Kaila Pianalto and Andrew Alspaugh [32] thoroughly reviews the literature on the clinically approved antifungals and the ones with novel modes of action. These include those agents targeting cell membranes, membrane-associated lipids, cell-wall synthesis inhibitors, fungal mitochondria, fungal sphingolipids, antifungal compounds with unknown mechanisms of action and repurposing clinically approved drugs for other conditions as antifungals agents.

The paper by Cecilia Li et al. [33] focuses on the phospholipase/inositol polyphosphate kinase pathway and why these enzymes represent suitable candidates for antifungal drug development. The pyrophosphate product is important for fungal fitness and pathogenicity. As the fungal pathway is significantly different than the mammalian counterpart, it provides opportunities for drug development.

The paper by Renata Azevedo et al. [34] focuses on an interesting aspect of antifungal drug development, which is to target fungal factors required for virulence instead of fungal factors required for growth. As discussed in the paper, this approach may significantly expand the repertoire of microbial targets, may preserve the host microbiome and may exert less selective pressure, which may result in a decrease of the development of resistant organisms.

The paper by Frazier Baker et al. [35] proposes the development of a quantitative model to predict the development of drug resistance in *Pneumocystis*. This model is particularly suited for such studies because of the limited therapeutic options. The model is based on the computational analysis of the identified amino acid mutations in the target protein to predict the protein’s resistance to inhibitor(s). The possibility to evaluate multiple simultaneous mutations represents a significant advantage compared to other methods that compare one mutation at a time. This model could potentially be applied to other proteins in other organisms, and thus it may provide significant insights into the resistance mechanisms in other fungi as well.

To conclude, I would like to thank each of the authors for their contribution in producing this Special Issue on novel antifungal drug discovery. Obviously, the papers included in this Special Issue represent only examples of the variety of new antifungal targets that are in development.

Finally, I wish to acknowledge the thorough work of the peer reviewers who contributed to improving the submitted manuscripts and I also would like to thank the staff members of the *JoF* Editorial Office, in particular Mr. Aimar Xiong, for their support.
